# Strength and Environmental Performance Evaluation of Weathered Hydrocarbon Contaminated Soil Treated with Modified Plantain Peels—A Low Carbon Remediation Solution

**DOI:** 10.3390/ma17205108

**Published:** 2024-10-19

**Authors:** Raphael B. Jumbo, Colin Booth, Samuel Abbey

**Affiliations:** 1 School of Engineering, College of Arts, Technology and Environment, University of the West of England, Bristol BS16 1QY, UK; raphael.jumbo@uwe.ac.uk (R.B.J.); colin.booth@uwe.ac.uk (C.B.); 2 Centre for Architecture and Built Environment Research (CABER), College of Arts, Technology and Environment, University of the West of England, Bristol BS16 1QY, UK

**Keywords:** low carbon, remediation, contamination, engineering, hydrocarbons, soil

## Abstract

This study investigated the structural and environmental recovery of weathered hydrocarbon-contaminated soils using low-carbon solutions and aimed to ascertain the suitability of the remediated soils for engineering purposes. 25% (*w*/*w*) of ground ripe (RPP) and unripe (UPP) waste plantain peels were each added to 1 kg weathered hydrocarbon-contaminated soil samples and monitored for 90 days. Biological, physicochemical, and engineering properties were analysed for all samples in triplicates. After 90 days of remediation, RPP and UPP nutrients degraded the mid-distillate hydrocarbon alkanes by 93% and 88%, while the heavier hydrocarbon alkanes were degraded by 83% and 85%, respectively. The polyaromatic hydrocarbons (PAHs) had 89% and 93% degradation for RPP and UPP-treated soils, respectively, while the natural attenuation sample had 28% degradation. The soil compressive strength increased by 16% and 19% for RPP and UPP-treated soils, respectively, whereas the natural attenuation soil compressive strength remained fairly constant. It was observed that the remediated soil cohesion, angles of internal friction, maximum dry density, and optimum moisture content all improved as the remediation proceeded, which subsequently showed that the remediation influenced the engineering properties of the contaminated soils. Therefore, the remediation of the contaminated soil improved the structural suitability of the soils.

## 1. Introduction

The recovery of weathered hydrocarbons contaminated soils is an urgent necessity to mitigate the adverse impacts of the pollution. Soil-based weathered hydrocarbon contamination is a serious environmental issue of global interest, impacting biodiversity, human health, soil use, and ecosystems [1,2,3]. Hydrocarbon pollution in soils undergo weathering and consequently alters the soil’s biological, physical, and chemical properties [4,5,6]. Studies on soils contaminated by weathered petroleum hydrocarbons have shown severe toxic impacts on plants and animals, including humans [3,7,8]. Hydrocarbons inhibit plant-microbe interactions and decrease microbes’ ability to digest organic substances that plants require as nutrition [9,10,11]. The medium (C10–C19) to heavy (C20–C40) molecular weight petroleum hydrocarbons in the soil can be bioavailable to the soil ecosystem [12,13] and humans, through contact and sorption, soil microbes, groundwater, or farm products [4,14]. Soil physical and chemical properties such as soil moisture content, organic and total carbons, total nitrogen, compressibility, and penetrability are affected by weathered hydrocarbon [15,16]. The medium and heavy molecular weight hydrocarbons can cause soil strength and water dynamics to change, making the soil more hydrophobic and subsequently negatively impacting the soil engineering qualities [17,18]. Salimnezhad et al. [19] and Oluremi and Adedokun [20], in their separate research, investigated the viability of oil-polluted soil for engineering construction. The researchers observed that the oil contamination influenced the critical engineering properties of soils. This corroborates with the research of Amir et al. [21] on the shear strength properties of oil-contaminated soils. The researchers concluded that shear strength decreased with increasing crude oil content. Interestingly, some of the abandoned, weathered hydrocarbon-contaminated sites were once targeted for engineering projects and urban land development for economic, social, and residential development. Therefore, addressing weathered hydrocarbon contamination in soils is crucial to both the structural and environmental recovery of the polluted sites.

The need for sustainable remediation of weathered hydrocarbons has led to the adoption of low-carbon bioremediation techniques. Low-carbon bioremediation techniques are biodegradation methods that use biological-based materials that emit less carbon dioxide [22,23]. Biological materials (also called biomaterials) that are cheap and readily available as biodegradable wastes can be suitably adopted for bioremediation [24,25]. These techniques, when compared to other soil remediation methods, are highly economical and ecologically low risk [26,27]. Additionally, they improve soil quality and tend to eliminate or reduce greenhouse gas emissions during and after remediation, tending towards sustainability [28,29]. However, for low-carbon techniques to be sustainable in remediating weathered hydrocarbons, the techniques should meet the present needs of remediating the contaminants from the soil without compromising the future needs required of the soil to meet agricultural, economic, environmental, engineering, and people-oriented targets. The emerging trends in sustainability in low carbon remediation techniques are tending towards increasing contaminants biodegradation rate, increasing soil biomass, reducing nutrient leaching, eliminating carbon footprints, improving soil ecology and quality, and using low-cost and low eco-risk biomaterials [29,30,31]. The use of low carbon stimulants, such as biochar and compost, for bioremediation yielded weathered hydrocarbon degradation efficiencies averaged at 60% degradation for not more than 50,000 mg/kg TPH within 150 days [32,33,34]. Besides bioremediation, low-carbon biomaterials have also been implicated in the improvement of soil engineering properties [12,16,29]. Engineering properties of soils are those properties that can be used for quantifying the physical and mechanical behaviour of soils, such as cohesion, angle of internal friction, and compressibility [35,36,37]. Investing these engineering properties in remediated weathered hydrocarbons contaminated soil would inform on the suitability of the remediated soil for projects in construction and other related purposes. Therefore, the aim of this research is to investigate the suitability of remediated weathered hydrocarbon-contaminated soil for engineering purposes using low-carbon biostimulation strategies.

## 2. Materials and Methods

Weathered hydrocarbons (crude oil) contaminated soil samples were taken from the top to 0.5 m depth for sampling using trowels and soil auger. This depth is typical for foundation depth ranges in the built environment [38,39]. The pristine soil was collected 25 m away from the contaminated sites to ensure minimal variation in soil type. The pristine soil and weathered hydrocarbon-contaminated soil were obtained from prominent hydrocarbon-contaminated sites in the Gokana Area (Latitude 4°43′19″ N, Longitude 7°27′9″ E), Rivers State, Nigeria. The soil samples were collected during the mid-dry season to ensure access to the contaminated field. The collected soil samples were transported in a sealed transparent polytetrafluoroethylene (PFTE) container to the laboratory. Low carbon biomaterials adopted for the biodegradation of hydrocarbon contaminants in the soil were ripe and unripe waste plantain peels, which are common biodegradable waste, and the adopted experimental approach ensured that the research tended towards sustainable reuse of waste. The plantain peels were washed and air dried for 24 h before oven drying at 100 °C for 24 h. The peels were manually crushed using a mini mortar and pestle and then sieved using a 2 mm aperture sieve as described by Oduje et al. [40]. The mesocosms experiments were conducted in triplicates, having untreated contaminated soil (for natural attenuation) and pristine soil as controls. 25% (*w*/*w*) of ground ripe and unripe plantain peels were each added to 1 kg of weathered hydrocarbons contaminated soil samples and monitored for 90 days. Research by Tian et al., Teng et al., Margesin et al., and Edema et al. showed above 70% hydrocarbon contaminants degradation with above 50% microbial recovery were observed from day 90 of remediation [9,12,26,33]. The application of ripe and unripe ground plantain peels to the contaminated soil was described by Song et al., Kuppusamy et al., and Teng et al. [1,7,12]. Biological, physicochemical, and engineering properties were analysed for the pristine soil and polluted and remediating soil samples.

### 2.1. Biological Properties

The total microbial count was determined using colony-forming unit (cfu) plate counting techniques. Soil suspensions were prepared by ten-fold serial dilutions of 1 g of soil using deionised water as diluents. 0.1 mL of dilutions were inoculated separately on nutrient agar plates in triplicates, thereafter, the inoculated plates were incubated at 37 °C for 24 h [9].

### 2.2. Physicochemical and Engineering Properties

The soil electrical conductivity and pH were determined with a Jenway pH meter (Chelmsford, Essex, UK) using 20 g of the air-dried soil sample and distilled water at 1:2 soil/water ratio. The mixture was shaken properly with a mechanical shaker and allowed to stand for 30 min before measurement. The exchangeable Sodium (Na) was determined by Flame Photometry techniques using 5 g air-dried soil (<2 mm) in a 50 mL centrifuge tube. The exchangeable aluminium was determined using 5 g of air-dried soil (passed through a 2 mm sieve) into a 50 mL centrifuge tube with 30 mL of KCl and Centrifuged at 2000 rpm for 15 min. The extract was analysed following the titrimetric standard method [41]. The organic carbon was determined using 1 g of soil in a 500 mL conical flask, with 10 mL of K_2_Cr_2_O_7_ and 20 mL of concentrated H_2_SO_4_ [42]. The soil petroleum hydrocarbon degradation was monitored using the method described by Risdon et al. [43]. The soil samples of petroleum hydrocarbons were extracted and analysed following the procedure outlined by Risdon et al. [43]. Soil hydrocarbon concentrations were identified and quantified utilising an Agilent (7000C) gas chromatograph (Santa Clara, CA, USA) connected to a Turbomass Gold mass spectrometer operating (Waltham, MA, USA) in positive ion mode at 70 eV. Standard solutions of alkanes (C8–C40) and PAHs (EPA 525 PAH Mix A) with concentrations between 1 and 5 μg/mL were used for external multi-level calibrations. Blank controls were analysed at regular intervals for every ten samples to ensure quality control.

The soil-saturated hydraulic conductivity was determined using the constant head permeameter techniques [44]. The standard proctor compaction test was used to determine the soil bulk density as described by the American Society for Testing Materials (ASTM) [45]. The soil was thoroughly mixed on the mixing tray and compacted into three layers; each layer was compacted with 25 blows of 2.5 kg rammer with a drop of 300/mm. The particle size distribution of the soil was determined by the Bouyoucos hydrometer method, according to Gavlak et al. [46]. The specific gravity of the soil sample was determined after the sample was oven-dried at 105 °C in 24 h. To remove air from the soil-water mixture, the setup was vacuum-pumped until all of the entrapped air had been removed. The soil moisture content was determined with 20 g of soil oven to dry at 105 °C [47]. A triaxial test for shear strength determination was carried out using the standard cylinder with dimensions of 100 mm diameter by 200 mm height. The compressive strength was determined after every 60 s for a strain between 6%, 12%, and every 2 min beyond 12%. The test was continued until an axial strain of 20% was reached when failure surfaces developed.

### 2.3. Statistical Analysis

Descriptive statistics were done using JMP Pro (version 16) and Microsoft Excel (Version 2111 Build 16.0.14701.20278). Descriptive statistical analysis carried out includes mean, standard deviation, and standard error. Standard error was used to evaluate the variability across the hydrocarbons and engineering properties, while the standard deviation was used to ascertain the variability within the other measured physicochemical properties of the samples. The mean was accessed across all measurements because the parameters were measured in triplicates.

## 3. Results and Discussion

### 3.1. Soil and Plantain Peels Characterisation

The characterisation of the experimental pristine soil, unripe (UPP) and ripe (RPP) ground plantain peels, contaminated soil (natural attenuation), and UPP- and RPP-treated contaminated soils were shown in Table 1 and Table 2.

The pristine soil was sandy loam, with a specific gravity of 2.79 ± 0.08 (Table 1). Across the contaminated and remediated soil samples, the specific gravity was fairly constant, ranging between 2.72 and 2.76 (Table 1), showing a slight decrease due to soil pollution. Holtz [48] and Prakash et al. [49] stated that typical values for soil-specific gravity suitable for engineering purposes range between 2.6 and 2.8. The pristine and contaminated soil pH was slightly acidic, which could be linked to the continuous acidic rainfall experienced in the Niger Delta, Nigeria [50]. The application of the ripe and unripe ground plantain peels with pH of 8.4 and 6, respectively (Table 2), slightly increased the soil pH from 5.5 to 5.9 and 5.7, respectively (Table 1). The introduction of the remediating bio-substrate into the contaminated soil increased the soil organic carbon (Table 1). This finding agreed with that of Wei et al. [51], the researchers stated that in low carbon biostimulation, added nutrients stimulate the soil microbial activities and increase organic carbon.

The pristine soil microbial content was ascertained through the heterotrophic bacterial content shown in Table 3.

From the table, the pristine soil had a fairly constant heterotrophic bacterial content (about 22 × 10^5^ cfu/g soil) from day 0 to 90. The hydrocarbon pollution led to a drastic decrease in the microbial content of the soil, having 0.153 × 10^5^ cfu/g soil (Table 3). The application of UPP and RPP nutrients to the contaminated soils increased the microbial content of the soil to 9650 × 10^5^ cfu/g soil and 19,500 × 10^5^ cfu/g soil, respectively, at day 90 (Table 3). The increase in microbial content could be linked to the provision of the necessary nutrients from UPP and RPP (Table 2), which are needed for the optimal functioning of the soil microbes and the possible use of the hydrocarbons as a source of food and energy by the microbes. The increment in microbial contents observed indicated low risk and low toxicity to the soil ecosystem and improved soil health [50,52] at the end of the experiment.

Soil-saturated hydraulic conductivity can be used to predict soil health; it governs water flux through the soil, and it is based on Darcy’s law [53]. Table 1 shows variations in the various soil samples. The pristine soil hydraulic conductivity was 0.0028 cm/s, whereas the polluted soil had 0.0004 cm/s. The presence of hydrocarbons reduced the water seepage through the soil, which subsequently reduced the soil porosity, making it difficult for water to drain through the weathered hydrocarbons polluted soils. After 90 days of remediation, there was a noticeable increase in the hydraulic conductivity of the soil (Table 1). The UPP- and RPP-treated soils had saturated hydraulic conductivity of 0.0016 cm/s and 0.0021 cm/s, respectively. Soil hydraulic conductivity contributes to understanding issues related to soil health, engineering, and naturally occurring earth-fill structures through evaluation of the subsurface water flow, seepage through and beneath earth-fill structures like dams or performance of soil drainage systems [54,55].

### 3.2. Efficiency in Hydrocarbon Remediation

**Figure 1:**
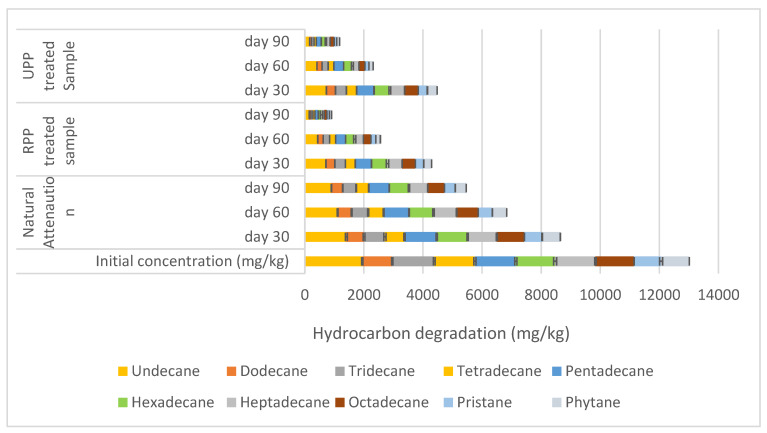
Degradation between undecane and phytane.

The hydrocarbon content in soils is a measure of the level of the hydrocarbons in the contaminated soils [19,29]. At the start of the experiment, the soil’s total hydrocarbon content was 13,500 mg/kg (Table 1) compared to the requirements of most global environmental agencies, which is prominently less than 500 mg/kg [56,57,58]. At day 90 of the remediation, the natural attenuation had 53% of the soil mid-distillates hydrocarbon alkanes (C11–C20) degraded, whereas the RPP and UPP had 93% and 88%, respectively (Figure 1). Similarly, 60% degradation was obtained by Wei et al. and Tian et al. when biochar and compost were used to degrade alkane hydrocarbons in soil over 150 days, showing a 1.8 mg/kg/day reduction in degradation rate when compared to Figure 1 [32,33]. The increased degradation rate observed in Figure 1 could be linked to the increment in heterotrophic bacteria observed in the RPP and UPP samples during the degradation of these mid-distillate hydrocarbons (Table 3). The heterotrophic bacteria prominently used the hydrocarbon alkanes as an energy and carbon source [58].

**Figure 2:**
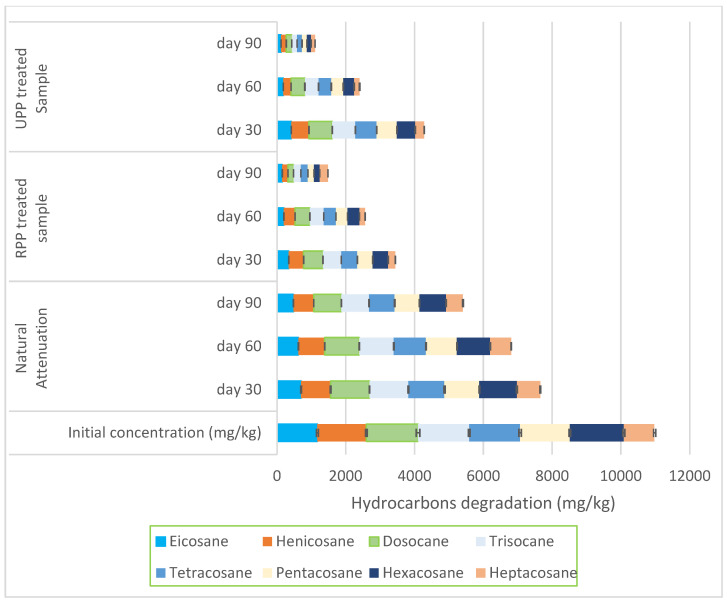
Degradation between eicosane and heptacosane.

**Figure 3:**
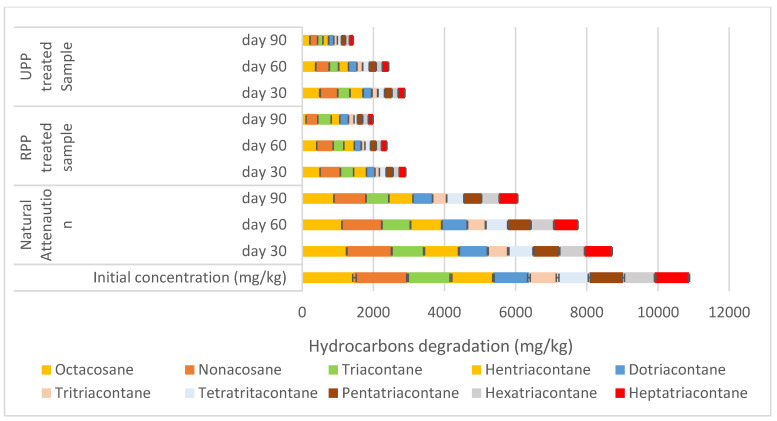
Degradation between octacosane and heptatriacontane.

The degradation between eicosane (C20) and heptacosane (C27) showed that RPP and UPP-treated samples were degraded by 84% and 85%, respectively, where the natural attenuation had 47% degradation (Figure 2). The degradation between octacosane (C28) and heptatriacontane (C37) is shown in Figure 3. From Figure 3, at day 90, RPP- and UPP-treated contaminated soils showed 82% and 85% degradation, while the natural attenuation had 44% degradation (Figure 3). As the remediation proceeded, it was observed that the degradation rate of the hydrocarbon alkanes (undecane–heptatriacontane) reduced as the molecular weight increased (Figure 1, Figure 2 and Figure 3). Furthermore, it was observed from Figure 1, Figure 2 and Figure 3 that whereas the RPP nutrient was very effective for remediating hydrocarbon alkanes of C11–C20, the UPP nutrient was more effective in enhancing the biodegradation of C20–C37 alkanes. These observed variations in hydrocarbon degradation could be linked to the varying nitrogen, phosphorus, and potassium values of RPP and UPP biostimulants (Table 2), which improved the production, stability, and activity of ALK plasmid responsible for synthesising encoding enzymes for degrading alkanes in various bacteria [59,60]. The findings of this research agreed with that of Omenna et al. [23] where the researchers stated that when microbes are enriched with limiting nutrients, the microbial capabilities are enhanced, resulting in improved reproduction and metabolic activities, which leads to the use of the contaminants as energy and food sources. In addition, the improvement in the RPP- and UPP-treated soil pores shown in the saturated hydraulic conductivity results (Table 1) could be another factor that enhanced the hydrocarbon degradation since it improved the circulation of oxygen in the aerobic process. Varjani and Upasani [61] stated that the improvement in soil aeration and nutrient availability enhances microbial degradation of contaminants in soil.

**Figure 4:**
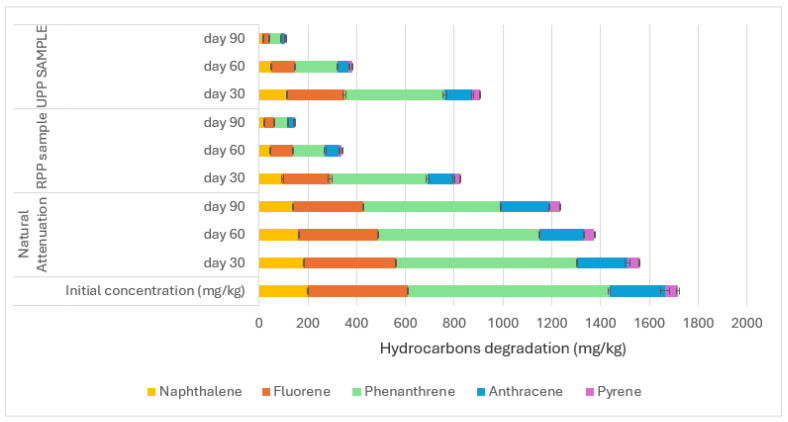
Degradation between naphthalene and pyrene.

**Figure 5:**
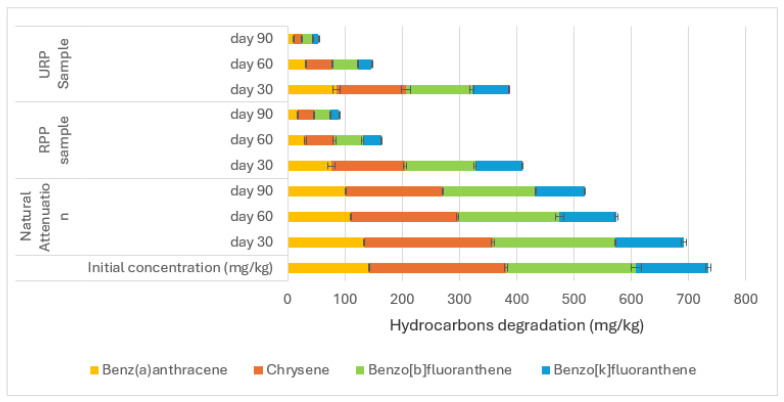
Degradation between benz(a)anthracene and benzo(k)fluoranthene.

**Figure 6:**
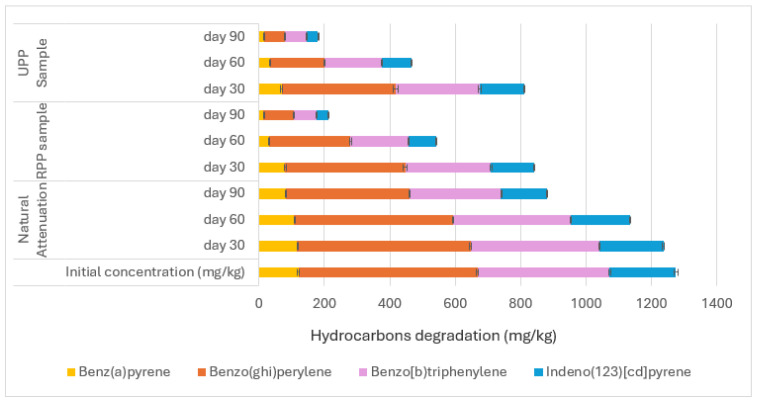
Degradation between benz(a) pyrene and indeno(123)[cd]pyrene.

The bioremediation of the polycyclic aromatic hydrocarbons (PAHs) is shown in Figure 4, Figure 5 and Figure 6.

The application of RPP and UPP in the contaminated soils led to more efficient degradation between naphthalene (C10) and pyrene (C16) when compared to the natural attenuation (Figure 4). The RPP- and UPP-treated soils showed 89% and 93% degradation of C10–C16 PAHs, respectively, compared to the natural attenuation, which had 28% degradation after 90 days of microbial activities on naphthalene (C10) to pyrene (C16) (Figure 4). The degradation of benz(a)anthracene (C18) to benzo(k)fluoranthene (C20) when RPP and UPP nutrients were applied to the contaminated soil showed 89% and 92% degradation compared to the natural attenuation, which had 30% degradation (Figure 5). Nutrients from RPP and UPP, such as nitrogen, phosphorus, and potassium (Table 2), are essential for the microbial production of ring-hydroxylating dioxygenases (RHDs), which are prominent enzymes for the initial catalysis of polycyclic aromatic hydrocarbons (PAHs) in bacteria [23,62]. After that, enzymes like ring-cleavage dioxygenases and dehydrogenases further break down the aromatic rings in the dihydrodiol, making the PAH intermediates very vulnerable to biodegradation [63,64]. The degradation of benz(a) pyrene to indeno(123)[cd]pyrene is shown in Figure 6. The RPP, UPP treated contaminated soil samples, and the natural attenuation samples had 83%, 86%, and 30% degradation, respectively (Figure 6). It was observed that the treatment of the soil with UPP was the most effective for PAH degradation, having led the RPP-treated soil with about an average of 3.3% (Figure 4, Figure 5 and Figure 6). This observed difference could be linked to the variation in the pH and nutrient quality of the UPP and RPP nutrients (Table 2). The results from Figure 4, Figure 5 and Figure 6 have shown that UPP and RPP are good biostimulants for remediating polycyclic aromatic hydrocarbons in soil compared to the application of exogenous microbes to degrade PAH contaminants in soil, which showed 60–70% efficiency [62,63,64]. The results from Figure 4, Figure 5 and Figure 6 agreed with the conclusions of Benyahia and Embaby [65] that substrates with varying pH, nitrogen, phosphorus, and potassium, when applied in the soils, stimulate the soil microbial abilities differently.

### 3.3. Engineering Suitability of the Remediated Soils

**Figure 7:**
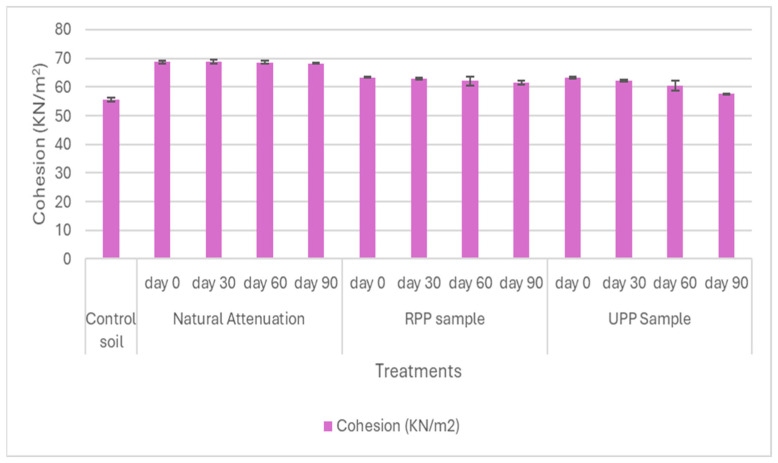
Cohesion.

**Figure 8:**
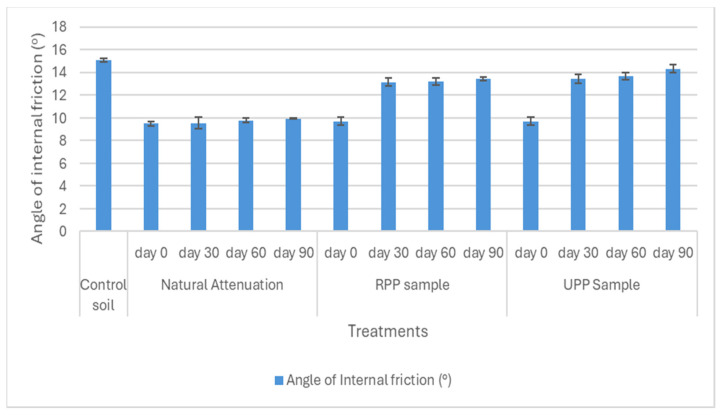
Angles of internal friction (°).

**Figure 9:**
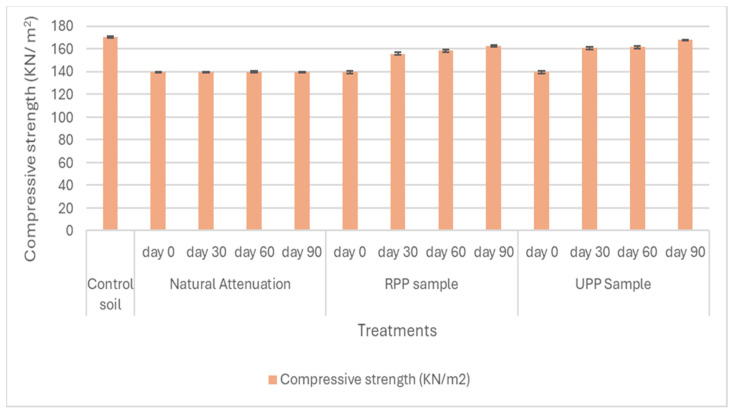
Compressive strength of samples.

The soil samples’ shear strength parameters, such as cohesion, angle of internal friction, and compressive strength, were shown in Figure 7, Figure 8 and Figure 9.

At day 0, the natural attenuation sample had the highest cohesion with 68.7 KN/m^2,^ while the RPP- and UPP-treated samples had 63 KN/m^2^, whereas the pristine soil cohesion was 55 KN/m^2^ (Figure 7). The higher cohesion observed in the natural attenuation sample could be associated with the stickiness of crude oil (hydrocarbons) in the soil, which prominently occupied the soil voids. Pauzi et al. [66] averred that when soils are compacted with pores occupied or closed, the soil cohesion tends to increase. After 90 days of remediation, it was observed that the RPP- and UPP-treated samples were 61 KN/m^2^ and 58 KN/m^2,^ respectively, while that of the natural attenuation was 68.2 KN/m^2^ showing a gradual reduction in cohesion as the hydrocarbons were degraded. The presence of the UPP and RPP in the remediated soil could also contribute to the soil cohesion having remediated the hydrocarbons by more than 85% (Figure 1, Figure 2, Figure 3, Figure 4, Figure 5 and Figure 6) at day 90, but the cohesion still exceeds that of the pristine soil, which is 55 KN/m^2^ (Figure 7). It was further observed that the UPP-treated soil had a reduced cohesion compared to the RPP-treated soils, which could be linked to the higher degradation of the heavier hydrocarbons in the soils treated with UPP (Figure 3, Figure 6 and Figure 7). Khosravi et al. [67] observed an increase in soil cohesion when gas oil content in soil is increased from 2% to 20%. Similarly, Karkusha and Kareem [68], in their research on the impact of freshly spilt fuel oil on soils, concluded that the fuel oil had impacts on soil cohesion.

The angle of internal friction was at 15° for the pristine soil, while for the weathered hydrocarbon soil (natural attenuation) at day 0, it was 9.5° (Figure 8). After 90 days of remediation, the RPP- and UPP-treated soil angle of internal friction increased to 13° and 14°, respectively (Figure 8). Salimnezhad et al. [19] stated that oil contamination in soil reduces the angle of internal friction. The soil compressive strength for the pristine soil and contaminated soil were 170 KN/m^2^ and 140 KN/m^2^, respectively (Figure 9). This observed considerable reduction in soil strength could be linked to the changes in the soil pore fluid properties, which had been acknowledged to impact soil strength properties [69]. Khamehchiyan et al. [70] and Salimnezhad et al. [19], in their separate research on the effect of oil contamination on soil strength, concluded that oil contamination reduces soil strength. However, after 90 days of remediation, the soil compressive strength increased by 16% and 19% for RPP- and UPP-treated soils, respectively, whereas the natural attenuation soil compressive strength remained fairly constant (Figure 9).

**Figure 10:**
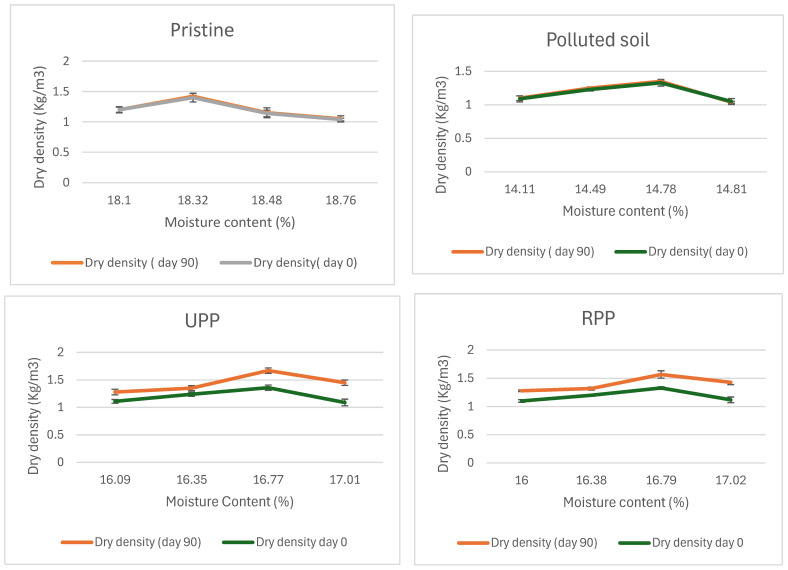
Dry density versus moisture content after remediation.

The optimum moisture content and maximum dry density for the pristine soil were fairly constant both at day 0 and 90 at 18.32% and 1.4 kg/m^3^ (Figure 10). The contaminated soil (natural attenuation) optimum moisture content remained the same at 14.78% for both days 0 and 90, while a slight variation was observed for the maximum dry density from 1.33 kg/m^3^ to 1.35 kg/m^3^ (Figure 10). Similar findings were made by Zahermand et al. [71] on the impact of crude oil on maximum dry density, and the researchers concluded that the lubricity of the contaminants decreased the friction between particles. The RPP- and UPP-treated soils had constant optimum moisture content both at day 0 and 90, having 16.79% and 16.77%, respectively. However, the maximum dry density of RPP and UPP increased by 18% and 23%, respectively, from days 0 and 90 (Figure 10). The observed variation in the dry density of the UPP- and RPP-treated samples can be linked to the presence of the ground plantain peels (for both ripe and unripe conditions), which were sieved to 2 mm, having slightly varying chemical composition, and the extent of the hydrocarbon contaminants remediation on the soil (Table 2 and Figure 1, Figure 2, Figure 3, Figure 4, Figure 5 and Figure 6). The variations in dry density and optimum moisture content give critical information on the effectiveness of the soil for engineering purposes [72].

## 4. Conclusions

The results from these investigations have shown that the UPP and RPP are very effective in remediating weathered hydrocarbons in soils. The RPP was more effective in remediating the mid-distillate hydrocarbon alkanes (C11–C20) in soils, having led UPP-treated soil by 5%. However, the UPP-treated soils led to the remediation of the heavier hydrocarbon alkanes (>C20) by 3%. The UPP-treated soil achieved more than 93% success in the remediation of PAHs, and the RPP-treated samples had 89% PAH degradation compared to the natural attenuation, which had less than 30% degradation. Overall, the remediation of the weathered hydrocarbon contaminants using RPP and UPP achieved almost 90% success. The soil shear strength was observed to improve as the remediation proceeds. After 90 days of remediation, the soil compressive strength increased by 16% and 19% for RPP- and UPP-treated soils, respectively, whereas the natural attenuation soil compressive strength remained fairly constant. The maximum dry density of RPP- and UPP-treated soils increased by 18% and 23%, respectively, from days 0 and 90.

The results from this study have shown that weathered hydrocarbon contamination impacts the soil engineering properties such as soil cohesion, angles of internal friction, compressive strength, maximum dry density, and optimum moisture content. The engineering value of the soil diminished due to weathered hydrocarbon contamination, which was evidenced by reduced compressive strength, angles of internal friction, maximum dry density, optimal moisture content, and increased soil cohesion. Subsequently, adequate remediation of the contaminants using RPP and UPP nutrients enhanced the soil potentials for engineering purposes by increasing the angles of internal friction, compressive strength, maximum dry density, and optimum moisture content and decreased the soil cohesion to a more appropriate value relevant for engineering purposes. The low carbon remediation of the weathered hydrocarbon contaminants with RPP and UPP biomaterials improved the suitability of the soils for engineering use. Therefore, the low-carbon bioremediation of the weathered hydrocarbons contaminated soil enhanced the structural and environmental recovery of the soils. Thus, low-carbon materials such as RPP and UPP biomaterials can be adopted for practical application in remediating hydrocarbon-contaminated sites using the approach developed in this research.

## Figures and Tables

**Table 1:** Physicochemical properties of Pristine soil, Polluted and Remediated Soils.

Parameters	Pristine Soil	Polluted	Remediated Soil with UPP	Remediated Soil with RPP
pH	5.5 ± 0.13	5.6 ± 0.12	5.7 ± 0.12	5.90 ± 0.11
Organic Carbon (%)	1.326 ± 0.02	0.780 ± 0.001	1.214 ± 0.02	1.204 ± 0.018
Organic Matter (%)	2.286 ± 0.07	1.345 ± 0.09	1.98 ± 0.088	2.102 ± 0.05
Exchangeable Sodium (mol/kg)	0.520 ± 0.001	0.026 ± 0.0001	0.209 ± 0.0001	0.254 ± 0.0012
Exchangeable Aluminium (mol/kg)	0.720 ± 0.0021	0.700 ± 0.002	0.720 ± 0.001	0.720 ± 0.002
Saturated Hydraulic Conductivity (cm/s)	0.0028 ± 0.0001	0.0004 ± 0.00001	0.0016± 0.0001	0.0021 ± 0.0001
Electrical Conductivity (μs/cm)	5.35 ± 0.21	24.60 ± 0.09	10.55 ± 0.011	10.6 ± 0.26
Specific gravity	2.79 ± 0.08	2.72 ± 0.091	2.76 ± 0.086	2.76 ± 0.075
Total petroleum hydrocarbon (mg/kg) (day 0)		13,502 ± 5.6	13,502 ± 5.4	13,501 ± 5.6
PSD	Sandy loam			
Sand (%)	58			
Silt (%)	27			
Clay (%)	15			

PSD—particle size distribution, UPP—unripe ground plantain peels and RPP—ripe ground plantain peels.

**Table 2:** Physicochemical Properties of UPP and RPP before Contamination.

Parameter	UPP	RPP
pH	6.00 ± 0.82	8.45 ± 0.01
Electrical conductivity (µS/cm)	6100.00 ± 81.65	5120.04 ± 0.01
Total dissolve solid (mg/L)	3050.00 ± 8.16	2503.00 ± 0.082
Salinity (mg/L)	2104.21 ± 0.01	1480.40 ± 0.08
Nitrogen (mg/L)	45.20 ± 0.01	29.08 ± 0.01
Potassium (mg/L)	20.00 ± 0.82	17.47 ± 0.01
Phosphorus (mg/L)	12.60 ± 0.08	8.40 ± 0.08

UPP = Waste unripe Plantain peels, RPP = Waste ripe plantain peels.

**Table 3:** Total Heterotrophic Bacterial (cfu/g soil) Population of Soil Samples.

Sample	Total Heterotrophic Bacteria (cfu/g soil) × 10^5^			
	Day 0	Day 30	Day 60	Day 90
Unpolluted	22	21.8	22.3	22.5
Polluted	0.153	0.229	0.314	0.36
Remediated UPP	0.165	39.4	788	9650
Remediated RPP	0.152	28.9	1420	19,500

UPP = Waste unripe ground Plantain peels, RPP = Waste ripe ground plantain peels.

## Data Availability

The original contributions presented in the study are included in the article, further inquiries can be directed to the corresponding author.
